# Chk2 Modulates Bmi1-Deficiency-Induced Renal Aging and Fibrosis via Oxidative Stress, DNA Damage, and p53/TGFβ1-Induced Epithelial-Mesenchymal Transition

**DOI:** 10.7150/ijbs.93598

**Published:** 2024-03-11

**Authors:** Jinhong Lu, Weiwei Sun, Boyang Liu, Jinge Zhang, Rong Wang, David Goltzman, Dengshun Miao

**Affiliations:** 1 The Research Center for Bone and Stem Cells, Department of Anatomy, Histology and Embryology, Nanjing Medical University, Nanjing, China.; 2 Calcium Research Laboratory, McGill University Health Centre and Department of Medicine, McGill University, Montreal, Quebec H4A 3J1, Canada.

**Keywords:** Bmi1, Chk2, aging-related renal fibrosis, p53, TGFβ1

## Abstract

Renal aging may lead to fibrosis and dysfunction, yet underlying mechanisms remain unclear. We explored whether deficiency of the Polycomb protein Bmi1 causes renal aging via DNA damage response (DDR) activation, inducing renal tubular epithelial cell (RTEC) senescence and epithelial-mesenchymal transition (EMT). Bmi1 knockout mice exhibited oxidative stress, DDR activation, RTEC senescence, senescence-associated secretory phenotype (SASP), and age-related fibrosis in kidneys. Bmi1 deficiency impaired renal structure and function, increasing serum creatinine/urea, reducing creatinine clearance, and decreasing cortical thickness and glomerular number. However, knockout of the serine-threonine kinase Chk2 alleviated these aging phenotypes. Transcriptomics identified transforming growth factor beta 1 (TGFβ1) upregulation in Bmi1-deficient RTECs, but TGFβ1 was downregulated upon Chk2 knockout. The tumor suppressor protein p53 transcriptionally activated TGFβ1, promoting EMT in RTECs. Bmi1 knockout or oxidative stress (induced with H_2_O_2_) increased TGFβ1 expression, and EMT in RTECs and was partly reversed by p53 inhibition. Together, Bmi1 deficiency causes oxidative stress and DDR-mediated RTEC senescence/SASP, thus activating p53 and TGFβ1 to induce EMT and age-related fibrosis. However, blocking DDR (via Chk2 knockout) or p53 ameliorates these changes. Our study reveals mechanisms whereby Bmi1 preserves renal structure and function during aging by suppressing DDR and p53/TGFβ1-mediated EMT. These pathways represent potential targets for detecting and attenuating age-related renal decline.

## Introduction

Renal fibrosis is a common consequence of aging-related kidney injuries and chronic kidney diseases (CKD), substantially impairing kidney function 1-3. Nevertheless, many of the underlying mechanisms leading to renal aging and fibrosis remain unclear. Furthermore, despite the need for safe and effective antifibrotic therapies, treatment options remain limited 4-6.

Renal tubular epithelial cell (RTEC) senescence is a key driver of renal fibrosis 7-10. Various factors trigger RTEC senescence, including DNA damage, mitochondrial dysfunction, inflammation, and oxidative stress 8, 11-15. Senescent RTECs accumulate with aging and kidney diseases, promoting fibrosis through releasing senescence-associated secretory phenotype (SASP) factors 7, 16, 17. RTEC senescence correlates with fibrosis progression 12, 18. Elucidating mechanisms governing RTEC senescence is therefore critical for developing antifibrotic therapies.

The Polycomb protein Bmi1 suppresses senescence by inhibiting the INK4a/ARF locus 19-22. Bmi1 also regulates mitochondrial function and a DNA damage response (DDR) 19-22. Bmi1 decline associates with escalating senescence 23-26, while Bmi1 deficiency causes oxidative stress, DDR activation, and premature senescence 20, 27, 28. Nevertheless, the precise molecular mechanisms linking Bmi1 deficiency to renal aging and fibrosis are not fully understood.

The serine-threonine kinase Chk2 transduces stress signals from DDR into senescence, apoptosis and other responses 29, 30. Sustained DDR induces senescence and fibrosis 11. Inhibiting Chk2 extends lifespan and alleviates pulmonary fibrosis in Bmi1-deficient mice 31. We previously showed Bmi1 loss causes kidney oxidative damage, DDR activation, premature aging, and fibrosis 32. However, whether Chk2 deletion mitigates Bmi1 deficiency-induced aging-related fibrosis by obstructing DDR signaling remains unknown.

This study explores whether Bmi1 deficiency leads to renal fibrosis through driving RTEC senescence/SASP, oxidative stress and DDR. It examines whether inhibiting Chk2 ameliorates aging-related fibrosis resulting from Bmi1 loss by mitigating these pathways, structural/functional decline, and activation of p53/TGFβ1/Smads signaling. Elucidating these mechanisms will aid in the development of senescence-targeted antifibrotic therapy.

## Materials and Methods

### Mice and Genotyping

Two mouse models were employed in this study. Bmi1 heterozygous (Bmi1^+/-^) mice were generously provided by Dr. Anton Berns from the Netherlands Cancer Institute. Chk2 heterozygous (Chk2^+/-^) C57BL/6J mice, offered by Professor Noboru Motoyama from the Longevity Sciences National Institute of Japan, were bred with Bmi1^+/-^ mice to generate double knockout (Bmi1^-/-^Chk2^-/-^) mice. Genotyping of mice was conducted using previously established protocols 20, 33. Genotyping information is provided in Supplementary Figure 2. The gene band display of Bmi1 gene knockout mice shows that Bmi1^+/+^ (wild-type) exhibits only one 687 bp band, Bmi1^-/-^ (homozygous) exhibits only one 399 bp band, and Bmi1^+/-^ (heterozygous) exhibits one 687 bp band and one 399 bp band. The gene band display of Chk2 knockout mice shows that Chk2^+/+^ (wild-type) exhibits only one 310 bp band, Chk2^-/-^ (homozygous) exhibits only one 424 bp band, and Chk2^+/-^ (heterozygous) exhibits one 310 bp band and one 424 bp band. Male mice were used in this study. All animal experiments strictly adhered to the guidelines of the Institute for Laboratory Animal Research of Nanjing Medical University and were approved by the Committee on the Ethics of Animal Experiments of Nanjing Medical University (Permit Number: IACUC-1802007).

### RNA Extraction and qRT-PCR

RNA extraction was performed using TRIzol reagent (#15596026; Ambion), and mRNA levels were quantified through qRT‐PCR as previously described 34. The sequence-specific primers are listed in Table 1.

### Western Blotting

Protein extraction from renal cortex or cells was carried out, followed by Western blotting as previously detailed 35. The primary antibodies used in this study were against Bmi1 (66161-1-lg; Proteintech), Chk2 (sc-5278; Santa Cruz Biotechnology), p-Chk2 (PA5-104715; Invitrogen), p16 (ab211542; Abcam), p53 (2527; Cell Signaling Technology), ac-p53 (ab75754; Abcam), p19 (sc-1665; Santa Cruz Biotechnology), p21 (sc-6246; Santa Cruz Biotechnology), PCNA (10205-2-AP; Proteintech), γ-H2A.X (9718; Cell Signaling Technology), ATM (2873; Cell Signaling Technology), p-ATM (sc-47739; Santa Cruz Biotechnology), p-p53 (9284, Cell Signaling Technology), TNF-α (ab1793; Abcam), IL-1β (ab9722; Abcam), NF-κB-p65 (8242; Cell Signaling Technology), p-NF-κB-p65 (3033; Cell Signaling Technology), IL-6 (sc-1265; Santa Cruz Biotechnology), TGFβ1 (21898-1-AP; Proteintech), TGFβR Ⅱ (ab186838; Abcam), Smad2 (sc-101153; Santa Cruz Biotechnology), p-Smad2 (3108, Cell Signaling Technology), Smad3 (sc-101154; Santa Cruz Biotechnology), p-Smad3 (ab529033; Abcam), Twist1 (sc-81417; Santa Cruz Biotechnology), Vimentin (ab92547; Abcam), Collagen I (1310-01; Southern Biotech), α-SMA (19245; Cell Signaling Technology), and E-Cadherin (ab76055; Abcam). β-actin (hrp6008; Proteintech) served as the loading control for both the cytoplasmic fraction and total cell protein. Immunoreactive bands were visualized using ECL chemiluminescence (P10300; New Cell & Molecular Biotech Co., Ltd) and analyzed with a chemiluminescence detection system (Bio-Rad, USA).

### Serum and Urine Biochemical Analyses

Serum creatinine (SCr) and blood urea nitrogen (BUN) levels were measured using a C011 Cr detection kit and a C013-2 BUN detection kit, respectively, as previously described 32, 33, following the manufacturer's instructions (Nanjing Jiancheng Bioengineering Institute). Urine creatinine (UCr) and urine albumin (UAL) levels were also measured using a C011 Cr detection kit and A028 UAL detection kit, respectively, according to the same protocols.

### Renal Color Doppler Ultrasonography

Prior to the procedure, a 24-hour fasting period was observed. Mice were anesthetized with inhaled ether, and their abdominal hair was removed. The peak systolic velocity (PSV) of the renal artery was measured using a small animal high-frequency ultrasound imaging system (Vevo 770, Atherosclerosis 199, Toronto, Canada) in accordance with previously described methods 36, 45. Pulse Doppler sampling was conducted on the renal artery.

### Histological Analyses

Fresh kidneys were fixed overnight in PLP fixative at 4°C, followed by standard histological processing 35. Tissues were dehydrated, embedded in paraffin, and sectioned into 5μm sections using a rotary microtome (Leica, Germany). These sections were subjected to tissue staining for total collagen and senescence-associated β-galactosidase (SA-β-gal) using established protocols 34.

### Transmission Electron Microscopy (TEM)

TEM was performed as previously described 32, 33. Fresh kidneys were bisected along the coronal plane, and tissue blocks not exceeding 1 mm^3^ were obtained from the cortical region of the kidney. The tissue blocks were fixed in 5% glutaraldehyde at 4°C for 24 hours, washed with PBS, post-fixed in 1% osmium tetroxide, dehydrated in acetone, embedded in epoxy resin, and cut into ultrathin sections approximately 800 nm thick. These sections were then examined and imaged using a transmission electron microscope (JEM-1010, JEOL, Japan).

### Immunohistochemical Staining

Immunohistochemical staining was conducted according to established methods 34, 36. Primary antibodies used included Bmi1 (66161-1-lg; Proteintech), Chk2 (sc-5278; Santa Cruz Biotechnology), p-Chk2 (PA5-104715; Invitrogen), p16 (ab211542; Abcam), p53 (2527; Cell Signaling Technology), p19 (sc-1665; Santa Cruz Biotechnology), p21 (sc-6246; Santa Cruz Biotechnology), PCNA (10205-2-AP; Proteintech), γ-H2A.X (9718; Cell Signaling Technology), 8-OHdG (ab48508; Abcam), p-ATM (sc-47739; Santa Cruz Biotechnology), TNF-α (ab1793; Abcam), IL-1β (ab9722; Abcam), NF-κB-p65 (8242; Cell Signaling Technology), IL-6 (sc-1265; Santa Cruz Biotechnology), CD3 (sc-20047; Santa Cruz Biotechnology), F4/80 (sc-377009; Santa Cruz Biotechnology), TGFβ1 (21898-1-AP; Proteintech), p-Smad2 (3108; Cell Signaling Technology), p-Smad3 (ab529033; Abcam), Twist1 (sc-81417; Santa Cruz Biotechnology), Vimentin (ab92547; Abcam), Collagen I (1310-01; Southern Biotech), α-SMA (19245; Cell Signaling Technology), and E-Cadherin (ab76055; Abcam). After washing, the secondary antibodies (horseradish peroxidase-labeled anti-mouse/rabbit IgG mixture; Gene Tech (Shanghai) Company Limited) were applied, followed by washing and DAB staining.

### Cell Cultures

Primary RTECs were isolated and cultured as previously described 8. The HK2 cells (human renal proximal tubular epithelial cell line) obtained from the American Type Culture Collection were cultured in the absence or presence of 10μM Pifithrin-α (PFTα, p53 inhibitor, #S2929, Selleck, USA) as previously outlined 37. The 293T cells (Shanghai Genechem Co., LTD) were cultured following the provided instructions.

### Transcriptome Sequencing and Data Analysis

Total RNA was extracted from primary RTECs derived from the cortex of 5-week-old WT, Bmi1^-/-^, and Bmi1^-/-^Chk2^-/-^ mice. RNA samples were quantified and assessed for quality using an Agilent 2100 bioanalyzer (Agilent, Agilent Technologies, Palo Alto, CA, USA). RNA sequencing was performed using Illumina NovaSeq 6000 to generate 150 bp paired-end reads. RNA sequencing and data analysis were conducted by Novogene Co. Ltd. using the Novogene cloud analysis platform. The resulting data are summarized in Table S1.

### Immunofluorescent (IF) Staining of Cells

IF staining of cells was performed following established methods 38, 39. Primary antibodies used included γ-H2A.X (9718; Cell Signaling Technology), p-ATM (sc-47739; Santa Cruz Biotechnology), p16 (ab211542; Abcam), Vimentin (ab92547; Abcam), α-SMA (19245; Cell Signaling Technology), E-Cadherin (ab76055; Abcam), Dylight 488‐conjugated secondary antibody (goat anti‐mouse IgG, BS10015; Bioworld Technology), Dylight 594‐conjugated secondary antibody (goat anti‐mouse IgG, BS10028; Bioworld Technology), Dylight594‐conjugated secondary antibody (goat anti‐rabbit IgG, BS10029; Bioworld Technology), and Dylight 488‐conjugated secondary antibody (goat anti‐rabbit IgG, A11008; Invitrogen). Cell proliferation was assessed using the EdU-594 Cell Proliferation Assay Kit (#C0078; Beyotime Institute of Biotechnology) following previously established protocols 38. To detect ROS levels, freshly harvested kidney tissues were frozen in liquid nitrogen and sectioned for staining (for live cells, staining was performed directly after removal of culture medium). Specimens were incubated with 10μM dihydroethidium (DHE, #S0063; Beyotime Institute of Biotechnology) at 37°C in the dark for 30 minutes, followed by staining with Hoechst (#C1027; Beyotime Institute of Biotechnology) at 37°C in the dark for another 30 minutes. The stained tissues were observed under a fluorescence microscope (Leica, Germany).

### Transfection of siRNA

HK2 cells were transfected with siRNA targeting human Bmi1 (sequence: 5′-AATGGACATACCTAATACT-3′), human Chk2 (sequence: 5′-GTAAGAAAGTAGCCATAAA-3′), or human p53 (sequence: 5′-GGAGTATTTGGATGACAGA-3′) for Bmi1/Chk2/p53 knockdown. A nontargeting negative control siRNA (sequence: 5′-UUCUCCGAACGUGUCACGUTT-3′) served as a control. All siRNA sequences were synthesized by Ribobio Co., located in Guangzhou, China. Transfection was carried out using Lipo2000 Transfection Reagent (Thermo Fisher Scientific Inc.), and protein analysis was conducted 48 hours after transfection.

### Chromatin Immunoprecipitation (ChIP)

ChIP assay was performed following the manufacturer's instructions (#17-610 Magna ChIPTM A; #17-408 EZ-Magna ChIPTM A, Millipore, USA). The p53 antibody was obtained from Cell Signaling Technology (#2527). ChIP primers were designed using Primer Premier 6 software: TGFβ1 sense: 5'-ATGCTTCCAGATGCCAGGTG-3' and anti-sense: 5'-TGTGTTATCCTCCTCCATGACC-3'. The relative binding of p53 to TGFβ1 was determined by qPCR.

### Dual Luciferase Reporter Gene Experiment

The p53 gene was cloned into the pcDNA3.1 vector (TranSheep Bio Co. Ltd.). Chimeric genes of the TGFβ1 promoter plasmids were constructed by inserting the luciferase gene upstream of the 5' flanking regions in a pGL4.1-basic vector (TranSheep Bio Co. Ltd.). Prior to transfection, 293T cells were seeded in 24-well culture plates and allowed to attach for 24 hours. For transfection, mixtures containing 1 μg each of pcDNA3.1-basic and pGL4.1-basic, overexpressed pcDNA3.1 and pGL4.1-basic, overexpressed pcDNA3.1 and pGL4.1-mutant (wherein the binding sequence "GGCTGCCCTGACATG" was mutated to "AAAAAAAAAAAAAAA"), overexpressed pcDNA3.1 and pGL4.1-promoter, overexpressed pcDNA3.1 and pGL4.1-mutant (with or without H_2_O_2_), and overexpressed pcDNA3.1 and pGL4.1-promoter (with or without H_2_O_2_) were co-transfected with Firefly luciferase - Renilla luciferase into the 293T cells using X-tremeGENE HP DNA Transfection Reagent (Roche Diagnostics Corp.). After 48 hours, the luciferase activity driven by the promoter was measured using a commercial kit (11402ES60; Yeasen, China). First, 200 μL of cell lysis solution was used to thoroughly lyse the cells, and then the mixture was centrifuged. Then, 20 μL of the supernatant was pipetted into a 96-well black/clear bottom plate, and 3 replicate wells were set up for each sample. Next, 100 μL of firefly luciferase reaction solution was added to the plate, and shaken to mix. The activity of firefly luciferase was monitored within 30 minutes. Following this, 100 μL of sea squirt luciferase reaction solution was added to the plate, and shaken to mix. The activity of sea squirt luciferase was then detected within 30 minutes. Finally, the data was analyzed according to the method provided in the instruction manual.

### Statistical Analysis

All data analyses were conducted using GraphPad Prism software (Version 8.0). Data from each group are presented as mean ± SEM. Statistical comparisons were performed using Student's t-test or two-way ANOVA for data comparison. P values were two-sided, and a value of less than 0.05 was considered statistically significant.

## Results

### Bmi1 Deficiency Activates Oxidative Stress, DDR, and Age-Related Fibrosis in the Kidney

**Figure 1:**
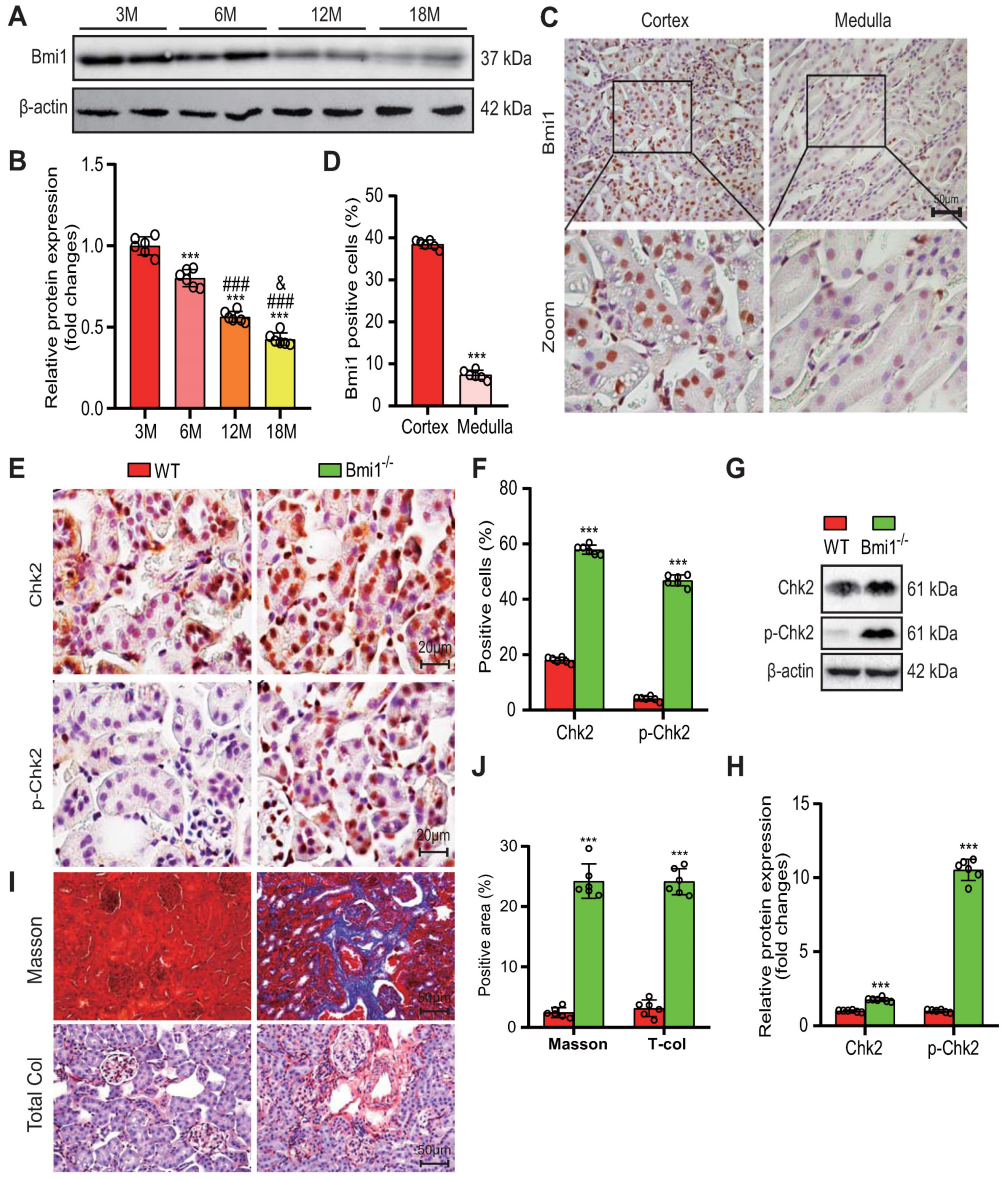
** Bmi1 deficiency activates oxidative stress and DDR, leading to age-related renal fibrosis. (A & B)** Western blotting analysis and statistical graphs showing protein expression levels of Bmi1 in the kidneys of 3, 6, 12, and 18-month-old WT mice. **(C & D)** Immunohistochemical staining and statistical graphs showing Bmi1 expression in the kidneys of 5-week-old WT mice. **(E & F)** Immunohistochemical staining and statistical graphs showing percentages of positive cells for Chk2 and p-Chk2.** (G & H)** Western blotting analysis and statistical graphs showing ac-p53 protein expression levels. **(I & J)** Micrographs showing Masson's staining (to visualize connective tissues), and Total Col (total collagen) staining, along with statistical graphs presenting the percentage of positive collagen fibers in the area. Each experiment was performed using 6 mice per group. Quantitative data are presented as mean ± SEM. *: *P*<0.05; ***: *P*<0.001, compared to 3-month-old wild-type mice (or Cortex group). ###: *P*<0.001, compared to 6-month-old wild-type mice. &: *P*<0.05, compared to 12-month-old wild-type mice.

In our study, we first aimed to examine Bmi1 expression during renal aging. We thus assessed the protein levels of Bmi1 in the kidneys of wild-type (WT) mice at different ages: 3, 6, 12, and 18 months. Remarkably, we observed a decline in Bmi1 protein levels as mice aged, as illustrated in Figure 1A and B. Immunohistochemical analysis further revealed that Bmi1 was predominantly expressed in RTECs of the cortex in WT mice, with minimal expression in the medulla (Figs. 1C and D). These findings suggested that Bmi1 might play a vital role in retarding the aging process of the renal cortex, particularly in cortical RTECs.

To investigate whether the absence of Bmi1 triggers oxidative stress and DDR in the kidneys, we employed various techniques, including TEM, DHE staining, Western blotting, immunohistochemical staining, and qRT-PCR to evaluate mitochondrial structure, levels of ROS, and markers of DDR in renal tissues. Comparative analysis between WT and Bmi1 knockout (Bmi1^-/-^) mouse kidney tissues revealed substantial increases in mitochondrial vacuolation, the percentage of DHE-positive areas, and the protein levels of DDR markers, including histone γ-H2A.X, the kinase ATM, p-ATM (phospho-ATM), Chk2, p-Chk2 (phospho-Chk2), p53, p-p53 (phospho-p53), and ac-p53 (acetyl-p53). Notably, Chk2, p-Chk2, γ-H2A.X, and 8-OHdG (8-hyroxyguanosine, a marker for endogenous oxidative damage to DNA) were predominantly expressed in cortical RTECs, with significantly elevated percentages of positive cells (Figs. 1F-I, Supplementary Figs. 1A-E), along with increased Chk2 mRNA expression levels in Bmi1^-/-^ mouse kidney tissues (Fig. 1E). These results collectively indicate that the absence of Bmi1 can activate oxidative stress and DDR mechanisms within the kidneys.

To assess whether Bmi1 deficiency contributes to renal aging, we examined various markers associated with aging in renal tissues. Comparing the kidney tissues of Bmi1^-/-^ mice to those of WT mice, we observed a significant increase in SA-β-gal activity (Supplementary Figs. 1B and C), primarily localized to cortical RTECs, along with significantly elevated protein levels of senescence markers p16, p19, p21, and p53 (Supplementary Figs. 1D and E). These findings support the conclusion that Bmi1 deficiency is associated with renal aging.

Finally, we investigated whether Bmi1 deficiency leads to renal fibrosis by evaluating fibrotic changes in renal tissues. In comparison to WT mouse kidney tissues, Masson's staining and total collagen staining clearly showed an increase in positive collagen fibers in Bmi1^-/-^ mouse kidney tissues (Figs. 1J -M). These results strongly indicate that Bmi1 deficiency is linked to the development of renal fibrosis.

### Chk2 Knockout Mitigates Renal Structural and Functional Damage Induced by Bmi1 Deficiency

**Figure 2:**
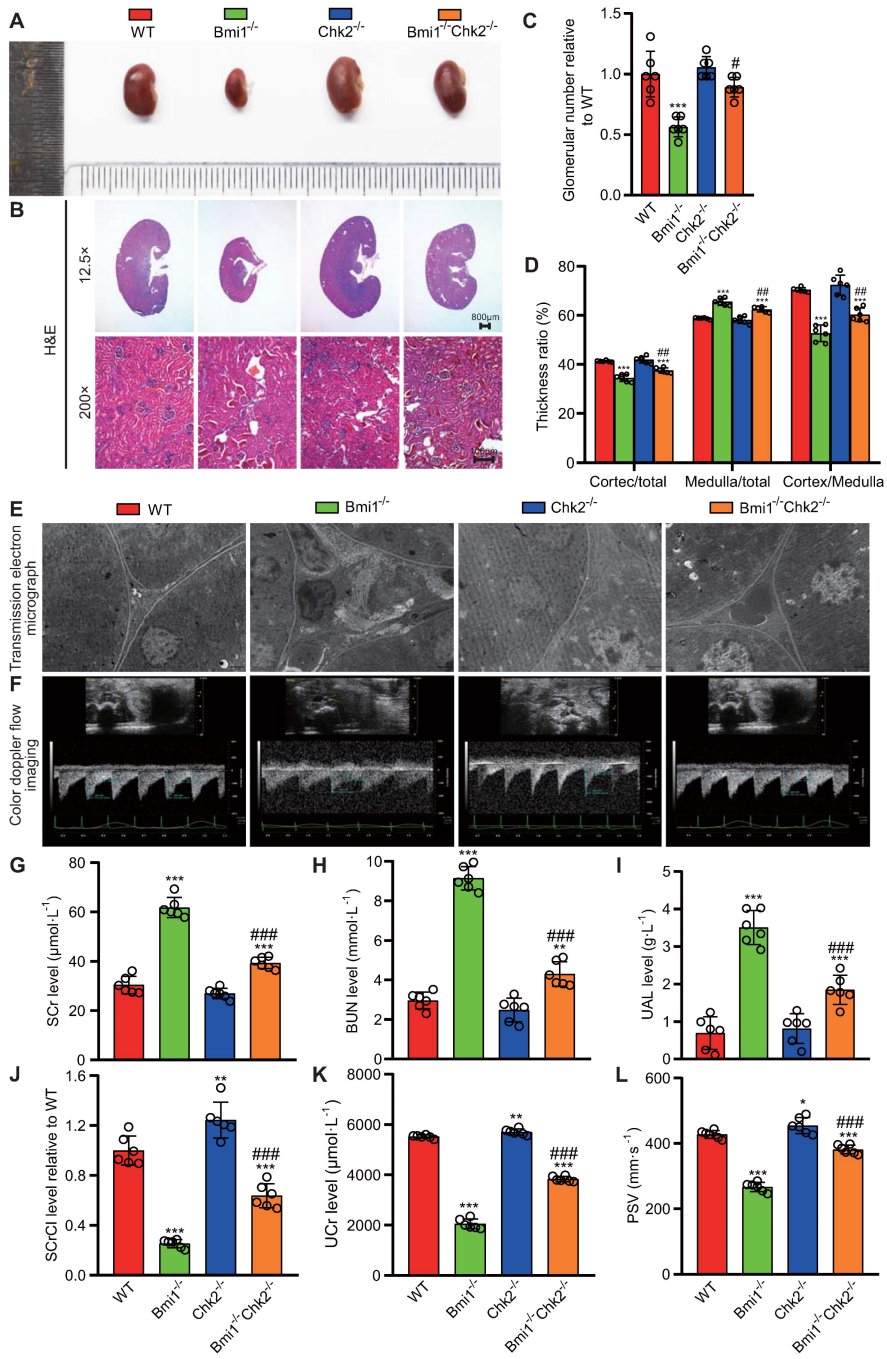
** Chk2 knockout ameliorates renal structural and functional damage caused by *Bmi1* deficiency. (A)** Representative images of kidneys from 5-week-old littermate WT, Bmi1^-/-^, Chk2^-/-^, and Bmi1^-/-^Chk2^-/-^ mice. **(B)** Representative H&E staining micrographs. **(C)** Quantification of glomerular number. **(D)** Statistical analysis of cortical/total thickness, medullary/total thickness, and cortical/medullary thickness ratio. **(E)** Representative TEM images. **(F)** Representative renal ultrasound images.** (G)** Statistical analysis of SCr. **(H)** Statistical analysis of BUN.** (I)** Statistical analysis of UAL.** (J)** Statistical analysis of SCrCl. **(K)** Statistical analysis of UCr. **(L)** Statistical analysis of PSV of blood flow in renal arteries. Each experiment was performed using 6 mice per group. Data are presented as mean ± SEM. *: *P*<0.05, **: *P*<0.01, ***: *P*<0.001, compared to 5-week-old WT mice; ##: *P*<0.01, ###: *P*<0.001, compared to 5-week-old Bmi1^-/-^ mice.

In this segment of our study, we sought to examine whether Chk2 knockout could ameliorate the renal structural damage resulting from Bmi1 deficiency, primarily by blocking DDR. We assessed renal structure through histological evaluation using H&E staining and TEM. Bmi1^-/-^ mouse kidneys exhibited several significant structural abnormalities compared to WT mouse kidneys. Notably, Bmi1^-/-^ mouse kidneys displayed a marked reduction in kidney volume (Figs. 2A and B), glomerular number (Figs. 2B and C), cortex thickness/total thickness ratio, and cortex thickness/medulla thickness ratio. Conversely, there was an increase in the medulla thickness/total thickness ratio (Figs. 2B and D), the number of interstitial myofibroblasts, and collagen fiber deposition within the renal interstitium (Fig. 2E). However, these structural alterations were partially restored upon Chk2 knockout (Figs. 2 A-E). To further investigate whether Chk2 knockout could mitigate the functional damage inflicted by Bmi1 deficiency, particularly by blocking DDR, we conducted blood and urine biochemical analyses and renal ultrasound assessments to evaluate kidney function. Bmi1^-/-^ mice demonstrated a significant increase in serum creatinine (SCr) levels (Fig. 2G), blood urea nitrogen (BUN) levels (Fig. 2H), and urinary albumin levels (UAL) (Fig. 2I). Additionally, they exhibited a significant decrease in the serum creatinine clearance rate (SCrCl) (Fig. 2J), urinary creatinine (UCr) levels (Fig. 2K), and peak systolic velocity (PSV) of blood flow in renal arteries (Figs. 2F and L) when compared to WT mouse kidneys. However, Chk2 knockout led to a partial restoration of these functional parameters (Figs. 2 F-L). Collectively, our findings indicate that Chk2 knockout has the potential to alleviate both the structural and functional damage induced by Bmi1 deficiency in the kidneys, primarily by inhibiting DDR.

### Chk2 Knockout Mitigates Bmi1 Deficiency-Induced Renal Oxidative Stress and DDR

**Figure 3:**
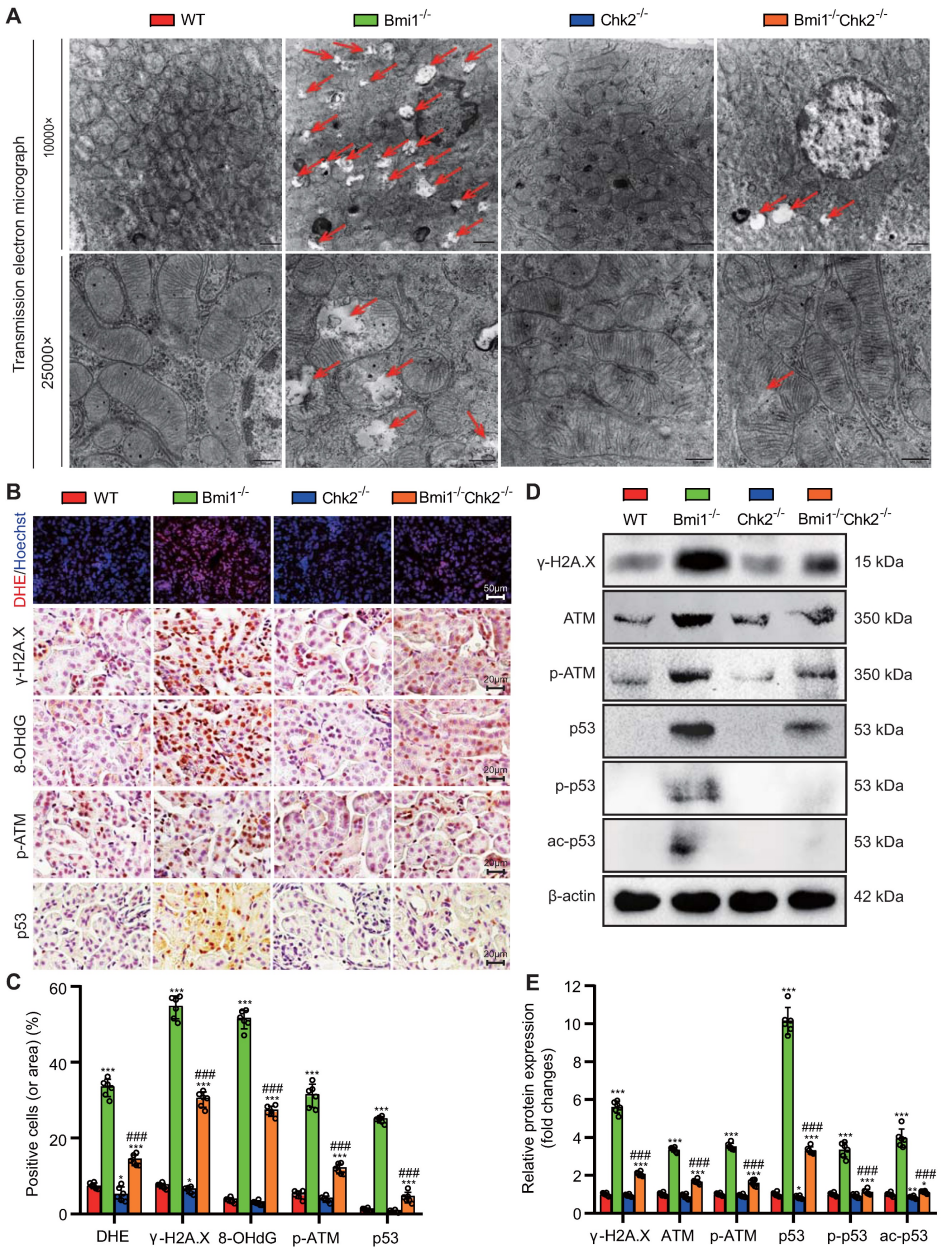
** Chk2 Knockout Mitigates Bmi1 Deficiency-Induced Renal Oxidative Stress and DDR. (A)** Representative TEM images showing mitochondrial vacuoles. **(B & C)** Immunohistochemistry images and quantitative analysis of DHE staining, γ-H2A.X, 8-OHdG, p-ATM, and p53 protein levels. **(D & E)** Western blotting analysis and quantification of γ-H2A.X, ATM, p-ATM, p53, p-p53, and ac-p53 protein levels. Six mice per group were used for the experiments. Data are presented as mean ± SEM. **: *P*<0.01, ***: *P*<0.001, compared to 5-week-old WT mice; ###: *P*<0.001, compared to 5-week-old Bmi1^-/-^ mice.

In this section of our investigation, we aimed to assess whether Chk2 knockout could ameliorate renal oxidative stress and the DDR triggered by Bmi1 deficiency. We employed a combination of techniques, including TEM, DHE staining, Western blotting, and immunohistochemical staining, to evaluate mitochondrial structure, ROS production, and DNA damage in kidney tissues. Comparing kidney tissues from Bmi1^-/-^ mice to those from Bmi1 and Chk2 double knockout mice, we observed significant improvements. Chk2 knockout in Bmi1 deficient mice led to a substantial reduction in the presence of mitochondrial vacuoles, the percentage of positive cells (or area) in DHE, and levels of γ-H2A.X, 8-OHdG, p-ATM, and p53 (Figs. 3A-C). Furthermore, Chk2 knockout in Bmi1 deficient mice resulted in reduced protein levels of γ-H2A.X, ATM, p-ATM, p53, p-p53, and ac-p53 (Figs. 3D and E). Taken together, these findings provide compelling evidence that Chk2 knockout can effectively ameliorate renal oxidative stress and the DDR induced by Bmi1 deficiency.

### Chk2 Knockout Alleviates Bmi1 Deficiency-Induced Renal Aging and SASP

**Figure 4:**
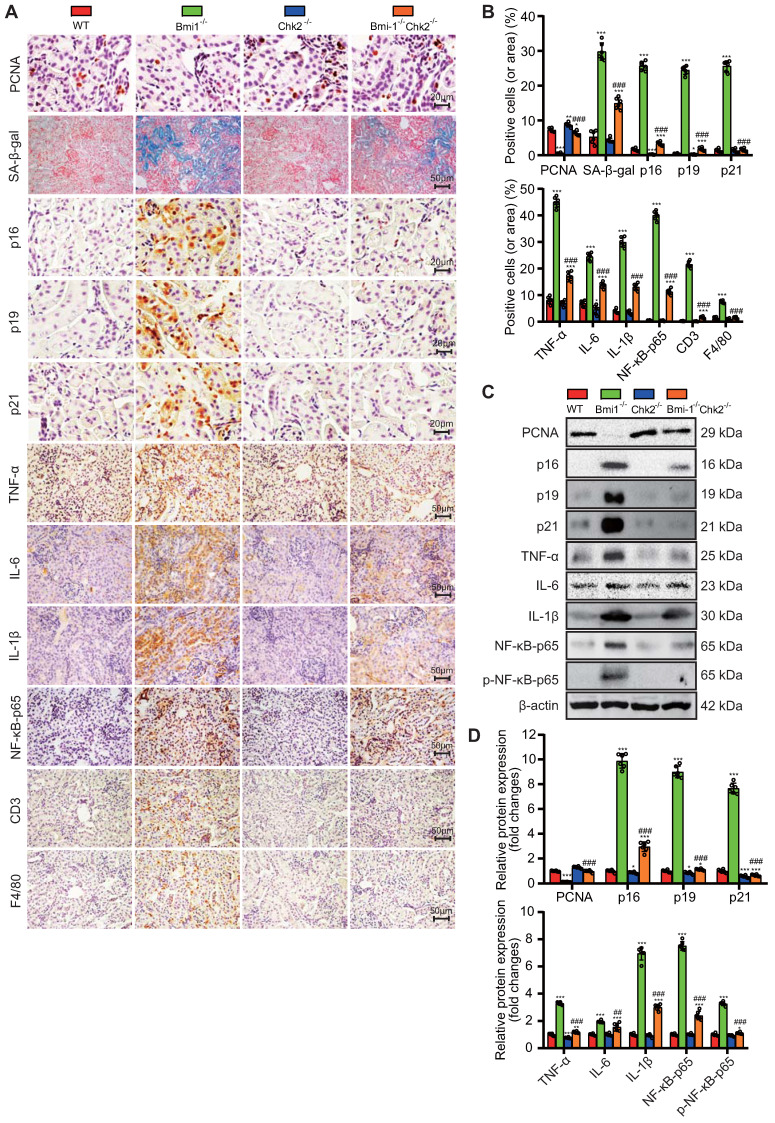
** Chk2 Knockout Alleviates Bmi1 Deficiency-Induced Renal Aging and SASP.** Paraffin-embedded kidney sections from 5-week-old littermate WT, Bmi1^-/-^, Chk2^-/-^, and Bmi1^-/-^Chk2^-/-^ mice were stained and analyzed. **(A & B)** Representative micrographs of PCNA, SA-β-gal histochemical staining, p16, p19, p21, TNF-α, IL-6, IL-1β, NF-κB-p65, CD3, F4/80 immunohistochemical staining, and the quantitative analysis of positive cell (area) percentage. **(C & D)** Representative Western blotting images and quantification of PCNA, p16, p19, p21, TNF-α, IL-6, IL-1β, NF-κB-p65, p-NF-κB-p65 protein expression levels. Six mice per group were used for the experiments. Data are presented as mean ± SEM. *: *P*<0.05, **: *P*<0.01, ***: *P*<0.001, compared to 5-week-old WT mice; ###: *P*<0.001, compared to 5-week-old Bmi1^-/-^ mice.

In this part of our investigation, we set out to determine whether Chk2 knockout could ameliorate renal aging induced by Bmi1 deficiency. We examined renal tissues using a combination of methods, including SA-β-gal staining, immunohistochemistry, and Western blotting. Chk2 knockout yielded significant improvements in the context of renal aging induced by Bmi1 deficiency. Specifically, Chk2 knockout resulted in a notable reduction in the percentage of SA-β-gal-positive areas and a decrease in the expression of aging-related proteins, including p16, p19 and p21, when compared to Bmi1^-/-^ mouse kidneys (Figs. 4A-D). Furthermore, Chk2 knockout in Bmi1 deficient mice led to an increase in the percentage of PCNA (proliferating cell nuclear antigen) -positive cells and elevated PCNA protein levels, indicating a positive impact on cell proliferation and repair mechanisms (Fig. 4A & B). Additionally, we investigated the effect of Chk2 knockout on Bmi1 deficiency-induced renal SASP. SASP markers were assessed in renal tissues, revealing that Bmi1^-/-^ mouse kidneys exhibited a significant increase in SASP markers, including the percentages of TNF-α, IL-6, IL-1β, NF-κB-p65, and p-NF-κB-p65-positive cells or area. Moreover, markers for T-cells (CD3) and macrophages (F4/80) also displayed an elevated percentage of positive cells in Bmi1^-/-^ mouse kidneys when compared to WT mouse kidneys (Figs. 4A-D). However, Chk2 knockout resulted in a significant reduction in the expression of SASP-related proteins, including TNF-α, IL-6, IL-1β, NF-κB-p65, and p-NF-κB-p65, as well as a decrease in the percentage of cells positive for the T cell co-receptor CD3 and the macrophage marker F4/80 in renal tissues, when compared to Bmi1^-/-^ mouse kidneys (Figs. 4A-D). These findings collectively demonstrate that Chk2 knockout has the potential to mitigate Bmi1 deficiency-induced renal aging and SASP.

### Chk2 Knockout Mitigates Aging-Related Renal Fibrosis Induced by Bmi1 Deficiency

**Figure 5:**
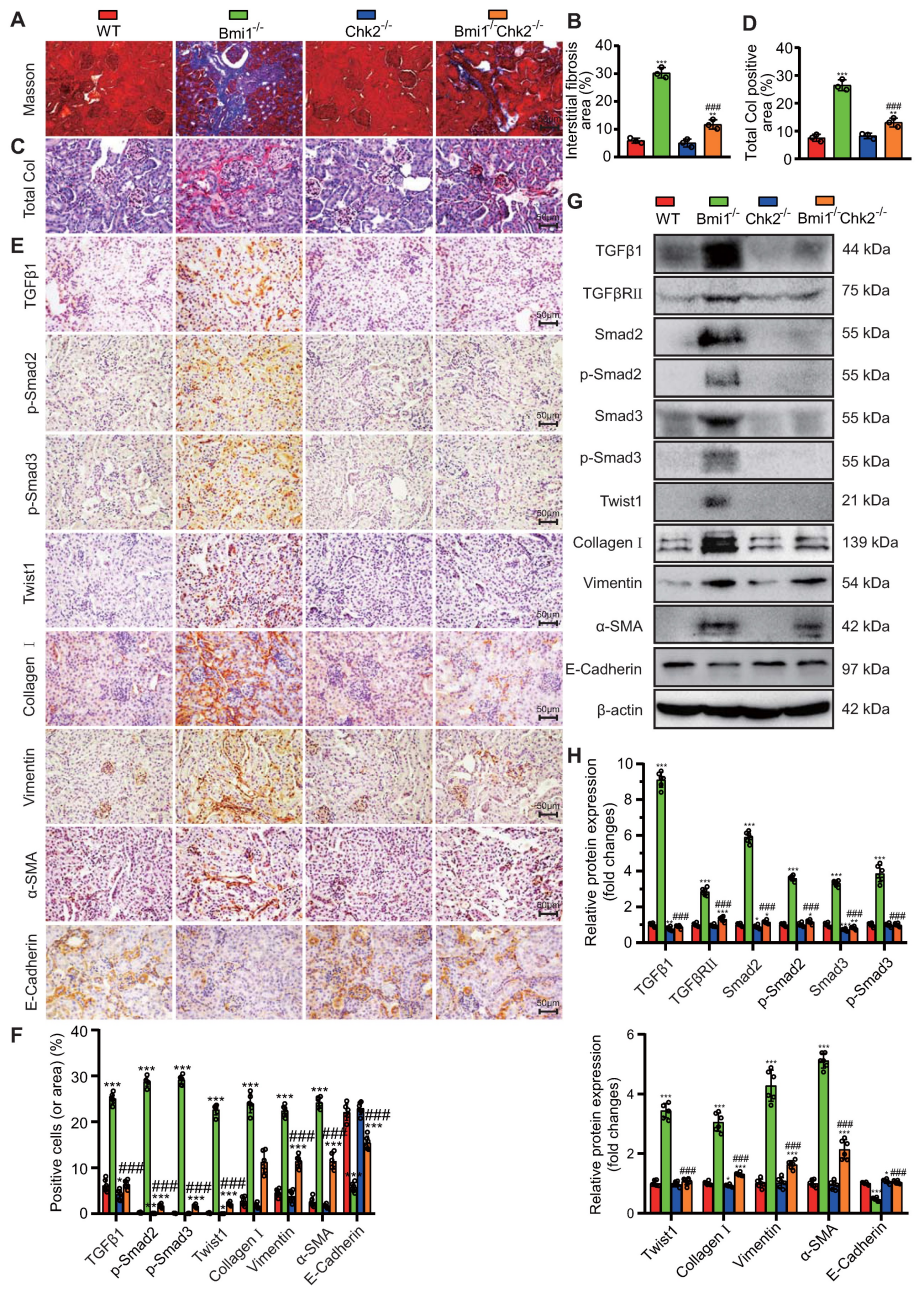
** Chk2 Knockout Mitigates Aging-Related Renal Fibrosis Induced by Bmi1 Deficiency. (A & B)** Representative micrographs of Masson's staining and quantification of interstitial fibrosis in kidney tissue sections from 5-week-old littermate WT, Bmi1^-/-^, Chk2^-/-^, and Bmi1^-/-^Chk2^-/-^ mice. **(C & D)** Representative micrographs of Total Col staining and quantification of total collagen positivity in kidney tissue sections. **(E & F)** Representative micrographs of immunohistochemical staining for TGFβ1, p-Smad2, p-Smad3, Twist1, Collagen I, Vimentin, α-SMA, and E-Cadherin, and quantification of positive cells or positive product area. **(G & H)** Representative Western blotting images and quantification of relative protein expression levels for TGFβ1, TGFβRⅡ, Smad2, p-Smad2, Smad3, p-Smad3, Twist1, Collagen I, Vimentin, α-SMA, and E-Cadherin. Each experiment was performed with 6 mice per group. Quantitative data are presented as mean ± SEM. *: *P* < 0.05, **: *P* < 0.01, ***: *P* < 0.001, compared to 5-week-old WT mice; ###: *P* < 0.001, compared to 5-week-old Bmi1^-/-^ mice.

In this section of our study, we aimed to explore whether Chk2 knockout could ameliorate aging-related renal fibrosis resulting from Bmi1 deficiency. We assessed renal fibrosis markers to evaluate the extent of fibrotic changes. Bmi1^-/-^ mouse kidney tissues exhibited a significant increase in total collagen staining area and collagen fiber area, as determined by Masson's staining, compared to WT mouse kidney tissues (Figs. 5A-D). Moreover, Bmi1^-/-^ mouse kidneys displayed a substantial elevation in the percentage of positive cells expressing markers associated with EMT and fibrosis. These markers included TGFβ1, p-Smad2, p-Smad3, and Twist1, as well as an increase in the percentage of positive areas for Collagen I, Vimentin, and α-SMA (smooth muscle actin). Additionally, there were elevated protein expression levels of TGFβRII and Smad2/3 in Bmi1^-/-^ mouse kidney tissues (Figs. 5E -H). Conversely, the percentage of positive area and protein expression levels of E-Cadherin, an epithelial marker, were significantly reduced in Bmi1^-/-^ mouse kidney tissues (Figs. 5E-H). However, Chk2 knockout intervention partially corrected these alterations observed in various fibrosis indicators induced by Bmi1 deficiency (Figs. 5A-H). These results collectively demonstrated that Chk2 knockout has the potential to attenuate aging-related renal fibrosis induced by Bmi1 deficiency, primarily by inhibiting the process of EMT in RTECs.

### Chk2 Knockout Mitigates Oxidative Stress, DNA Damage, Cellular Senescence, and EMT Induced by Bmi1 Deficiency in RTECs

**Figure 6:**
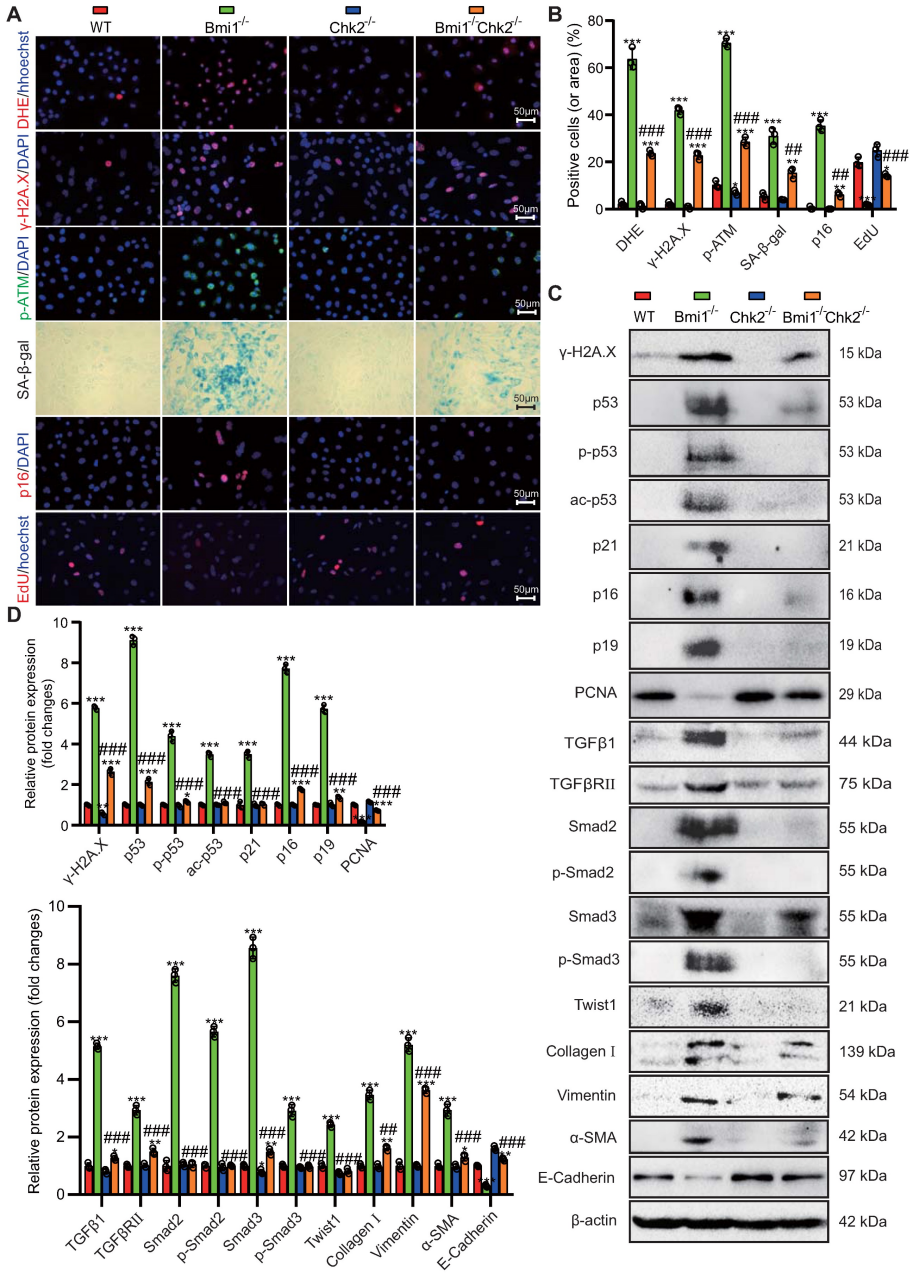
** Chk2 Knockout Mitigates Oxidative Stress, DNA Damage, Cellular Senescence, and EMT Induced by Bmi1 Deficiency in RTECs. (A & B)** Representative micrographs and quantification of DHE staining, γ-H2A.X, p-ATM, p16, EdU immunocytochemistry, and SA-β-gal staining in primary RTECs from the renal cortex of WT, Bmi1^-/-^, Chk2^-/-^, and Bmi1^-/-^Chk2^-/-^ mice; **(C & D)** Representative Western blotting and quantification of γ-H2A.X, p53, p-p53, ac-p53, p21, p16, p19, PCNA, TGFβ1, TGFβRⅡ, Smad2, p-Smad2, Smad3, p-Smad3, Twist1, Collagen I, Vimentin, α-SMA, and E-Cadherin protein expression levels in RTECs. Three biological replicates were used per experiment. *: *P*<0.05, **: *P*<0.01, ***:* P*<0.001, compared to 5-week-old WT RTECs; ##: *P*<0.01, ###: *P*<0.001, compared to 5-week-old Bmi1^-/-^ RTECs.

To delve deeper into the potential of Chk2 knockout to counteract oxidative stress, DNA damage, cellular senescence, and EMT provoked by Bmi1 deficiency, we conducted experiments using primary RTECs isolated from the renal cortex of 5-week-old littermate WT, Bmi1^-/-^, Chk2^-/-^, and Bmi1^-/-^Chk2^-/-^ mice. We employed various techniques, including DHE staining, IF staining, SA-β-gal staining, EdU incorporation, and Western blotting analysis, to assess ROS levels, DNA damage, proliferation, and senescence markers in RTECs. In Bmi1^-/-^ RTECs, we observed a significant increase in the percentage of DHE levels, γ-H2A.X, p-ATM, SA-β-gal staining, p16-positive cells or area (Figs. 6A & B), as well as an increase in γ-H2A.X, p53, p-p53, ac-p53, p21, p16, and p19 protein levels (Figs. 6C & D). Conversely, the percentage of EdU-positive cells and the expression of PCNA protein were significantly decreased compared to WT RTECs. Notably, Chk2 knockout partially restored these markers to varying degrees (Figs. 6A-D), suggesting that Chk2 knockout can attenuate the oxidative stress, DNA damage, and cellular senescence triggered by Bmi1 deficiency in RTECs.

To ascertain whether Bmi1 deficiency activates the TGFβ1/Smads signaling pathway in RTECs and whether Chk2 knockout has a regulatory role in EMT, we examined the expression levels of markers associated with the TGFβ1/Smads signaling pathway and EMT. In Bmi1^-/-^ RTECs, there was a substantial upregulation in the expression of TGFβ1, TGFβRⅡ, Smad2, p-Smad2, Smad3, p-Smad3, Twist1, Collagen I, Vimentin, and α-SMA proteins compared to WT RTECs. Conversely, in Bmi1^-/-^Chk2^-/-^ RTECs, these markers were downregulated when compared to Bmi1^-/-^ RTECs.

Additionally, the E-Cadherin protein expression level was significantly reduced in Bmi1^-/-^ RTECs compared to WT RTECs and was increased in Bmi1^-/-^Chk2^-/-^ RTECs when compared to Bmi1^-/-^ RTECs (Figs. 6C & D). These findings suggest that Bmi1 deficiency activates the TGFβ1/Smads signaling pathway, while Chk2 knockout has a modulatory effect on the EMT process in RTECs.

### Identification of Key Regulators of RTEC Mesenchymal Transition Modulated by Bmi1 Deficiency Activation and Chk2 Knockout Inhibition

**Figure 7:**
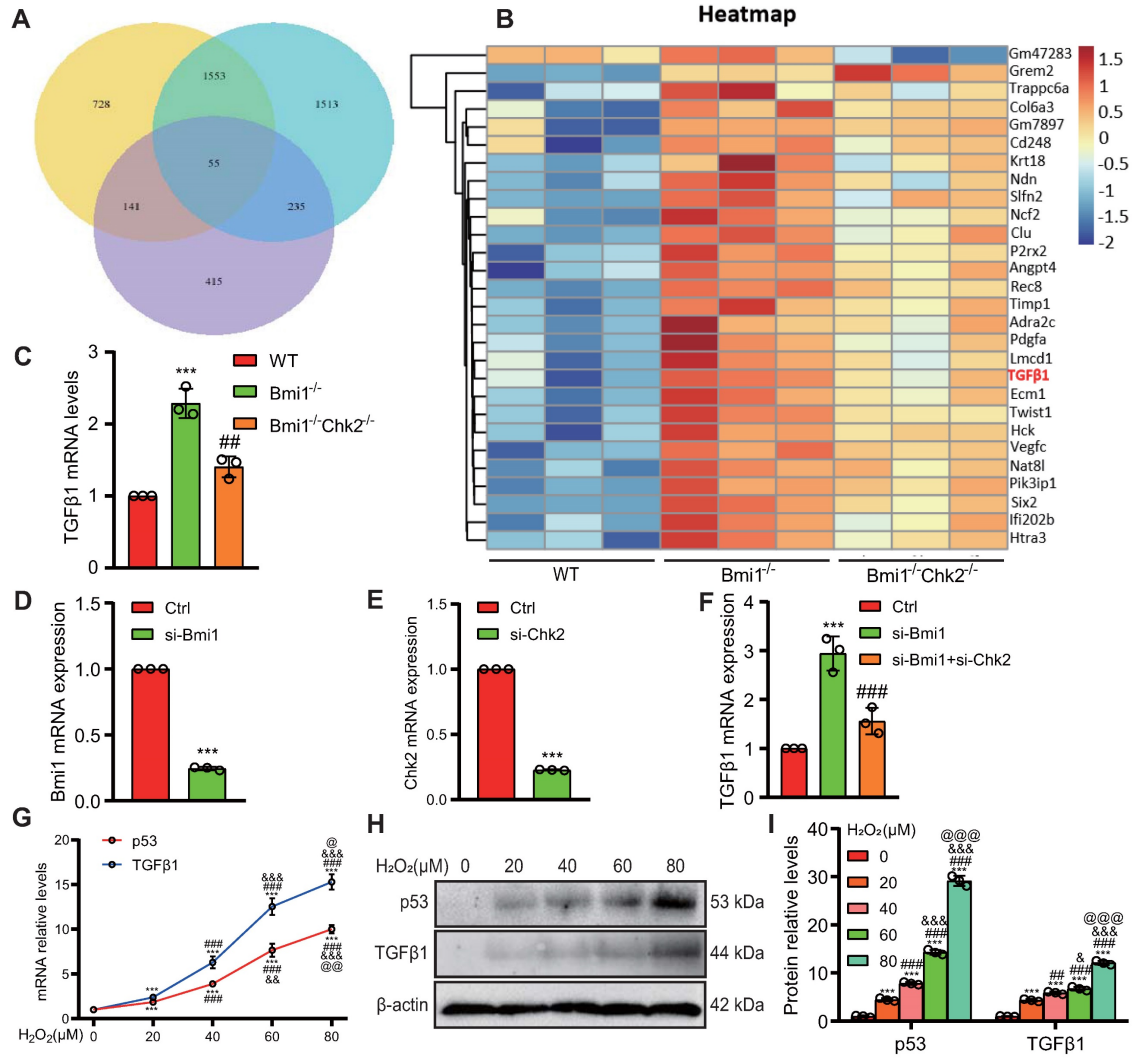
** Identification of Key Regulators of RTEC Mesenchymal Transition Modulated by Bmi1 Deficiency Activation and *Chk2* Knockout Inhibition. (A)** Venn diagram of RNA-seq results from primary cortical RTECs isolated from the kidneys of 5-week-old WT, Bmi1^-/-^ and Bmi1^-/-^Chk2^-/-^ mice, and **(B)** Heatmap of 28 differentially expressed genes that are upregulated in Bmi1^-/-^ RTECs and downregulated in Bmi1^-/-^Chk2^-/-^ RTECs. **(C)** The mRNA expression levels of TGFβ1 were validated by qRT-PCR in RTECs from the three genotypes. **(D)** The interference efficiency of si-Bmi1 in HK2 cells was detected 48 hours post-transfection and **(E)** the interference efficiency of si-Chk2 in HK2 cells was detected 48 hours post-transfection. **(F)** The mRNA levels of TGFβ1 were measured 48 hours after transfection with control, si-Bmi1, si-Chk2, or si-Bmi1+si-Chk2 in HK2 cells. HK2 cells were stimulated with gradient concentrations of H_2_O_2_, and **(G)** the mRNA expression levels of p53 and TGFβ1 were detected, along with **(H)** protein expression levels determined by Western blotting. **(I) S**tatistical analysis of the relative protein expression levels of p53 and TGFβ1. Three biological replicates were used per experiment. ***: *P* < 0.001, compared to HK2 cells or WT RTECs or the control group treated with 0 μM H_2_O_2_; ##: *P* < 0.01, ###: *P* < 0.001, compared to HK2 cells or Bmi1^-/-^ RTECs or si-Bmi1 cells treated with 20 μM H_2_O_2_; &: *P* < 0.05, &&: *P* < 0.01, &&&: *P* < 0.001, compared to HK2 cells treated with 40 μM H_2_O_2_; @: *P* < 0.05, @@: *P* < 0.01, @@@: *P* < 0.001, compared to HK2 cells treated with 60 μM H_2_O_2_.

To uncover pivotal molecules governing the mesenchymal transition of RTECs, which are upregulated by Bmi1 deficiency and downregulated by Chk2 knockout, we isolated mRNA from primary cortical RTECs obtained from the kidneys of 5-week-old mice. Employing transcriptome sequencing (The assigned accession of the submission is: CRA013740, and is publicly accessible at https://ngdc.cncb.ac.cn/gsa) and subsequent bioinformatics analysis, we identified a total of 55 genes that exhibited significant differential expression among the three genotypes of RTECs (Fig. 7A) (genes were selected as significantly differentially expressed with criteria of padj ≤ 0.05 and | log2 (fold change) | ≥ 1). Among these differentially expressed genes, 28 genes were upregulated in Bmi1^-/-^ RTECs and downregulated in Bmi1^-/-^Chk2^-/-^ RTECs. Notably, one of these identified genes was the profibrotic factor TGFβ1 (Fig. 7B). To validate these findings, we performed qRT-PCR analysis of TGFβ1 mRNA expression levels in primary cortical RTECs from 5-week-old WT, Bmi1^-/-^, and Bmi1^-/-^Chk2^-/-^ mice, confirming the consistency between qRT-PCR and sequencing results (Fig. 7C). Furthermore, we employed siRNA to knock down Bmi1 and/or Chk2 in HK2 cells and assessed TGFβ1 mRNA expression levels using qRT-PCR. The results demonstrated that TGFβ1 mRNA expression was significantly elevated in Bmi1 knockdown HK2 cells compared to the control group, while it was significantly reduced in HK2 cells with double knockdown of Bmi1 and Chk2 when compared to cells with single Bmi1 knockdown (Figs. 7D - F).

To mimic the oxidative stress-induced microenvironment and cellular senescence observed in Bmi1-deficient mice, we exposed HK2 cells to varying concentrations of H_2_O_2_ (0, 20, 40, 60, 80 μM) for 24 hours and examined changes in TGFβ1 expression as well as the key effector molecule p53, which regulates cellular senescence and DDR pathways, using Western blotting and qRT-PCR. The results indicated a significant upregulation of both TGFβ1 and p53 mRNA (Fig. 7G) and protein expression levels (Figs. 7H & I) with increasing H_2_O_2_ concentrations. These findings suggest that p53-mediated regulation of TGFβ1 may play a pivotal role in the Bmi1-deficiency-induced EMT of RTECs, and that Chk2 knockout may modulate this process by inhibiting TGFβ1 expression.

### Transcriptional Activation of TGFβ1 by p53

**Figure 8:**
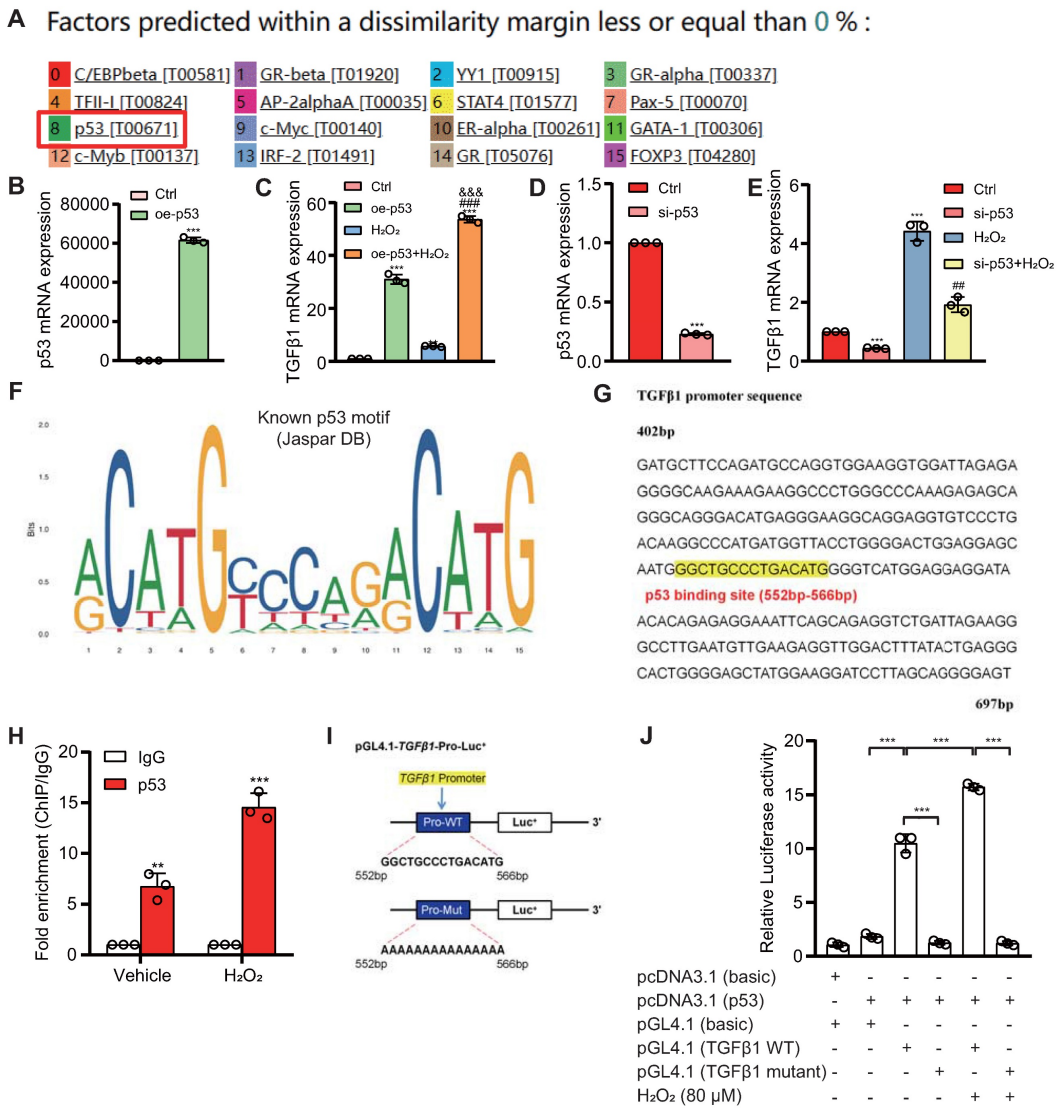
** Transcriptional upregulation of TGFβ1 by p53. (A)** Predicted upstream transcription factors of TGFβ1 from the PROMO database (https://alggen.lsi.upc.es/cgi-bin/promo_v3/promo/promoinit.cgi?dirDB=TF_8.3); **(B)** Transfection efficiency of oe-p53 plasmid in HK2 cells detected 48 hours post-transfection;** (C)** mRNA levels of TGFβ1 detected 48 hours after transfection of oe-p53 plasmid in HK2 cells with or without H_2_O_2_ treatment for 24 hours; **(D)** Transfection efficiency of si-p53 in HK2 cells detected 48 hours post-transfection;** (E)** mRNA levels of TGFβ1 detected 48 hours after transfection of si-p53 in HK2 cells with or without H_2_O_2_ treatment for 24 hours; **(F & G)** Predicted p53 binding site (highlighted in yellow) in the TGFβ1 promoter region;** (H)** Enrichment of TGFβ1 in p53 ChIP detected by qRT-PCR; **(I)** Schematic representation of the luciferase reporter gene plasmid containing the TGFβ1 promoter region; **(J)** Statistical analysis of luciferase activity. Three biological replicates were used per experiment. **: *P* < 0.01, ***: *P* < 0.001, compared to the control group; ##: *P* < 0.01, ###: *P* < 0.001, compared to oe-p53 or si-p53 cells; &&&: *P* < 0.001, compared to cells treated with H_2_O_2_ alone.

To investigate whether p53 can directly activate TGFβ1 expression at the transcriptional level, we utilized databases to predict potential upstream transcription factors of TGFβ1 and identified p53 among them (Fig. 8A). We designed a eukaryotic overexpression plasmid for p53 and a specific siRNA targeting p53, which we then transfected into HK2 cells. qRT-PCR analysis revealed that, compared to the control group, p53 overexpression significantly increased the expression of TGFβ1 mRNA in HK2 cells, with this effect being more pronounced when cells were exposed to 80 μM H_2_O_2_ (Figs. 8B & C). Conversely, p53 knockdown led to a significant decrease in TGFβ1 mRNA expression in HK2 cells, compared to both the control group and the group treated with 80 μM H_2_O_2_ (Figs. 8D & E). Through bioinformatics analysis, we identified a potential binding site "GGCTGCCCTGACATG" for p53 within the promoter region of TGFβ1 (Figs. 8F & G). To demonstrate the physical binding of p53 to this predicted site within the TGFβ1 gene promoter region, we designed specific primers targeting the p53 binding site and performed ChIP-qPCR. The results confirmed that p53 directly bound to the predicted binding site within the TGFβ1 gene promoter region, and this binding affinity was further enhanced under H_2_O_2_ treatment conditions (Fig. 8H). To assess whether the physical binding of p53 to the TGFβ1 gene promoter region has a biologically relevant impact on transcriptional regulation, we constructed luciferase reporter gene plasmids containing the p53 binding site within the TGFβ1 promoter region, as well as mutant plasmids (Fig. 8I). These plasmids were co-transfected into 293T cells, and luciferase activity was measured 48 hours later. The results demonstrated that, compared to the control plasmid group, luciferase activity significantly increased in the group transfected with both pcDNA3.1-p53 and pGL4.1-TGFβ1 plasmids, and this transcriptional activity was further enhanced by H_2_O_2_ treatment. In contrast, no significant increase in luciferase activity was observed in the group transfected with pcDNA3.1-p53 and pGL4.1-TGFβ1-mutant plasmids, even under H_2_O_2_ stimulation (Fig. 8J). These findings indicate that p53 can directly bind to a specific site within the TGFβ1 gene promoter region, leading to the transcriptional upregulation of TGFβ1 expression in HK2 cells.

### Activation of the p53/TGFβ1 Signaling Pathway Promotes EMT

**Figure 9:**
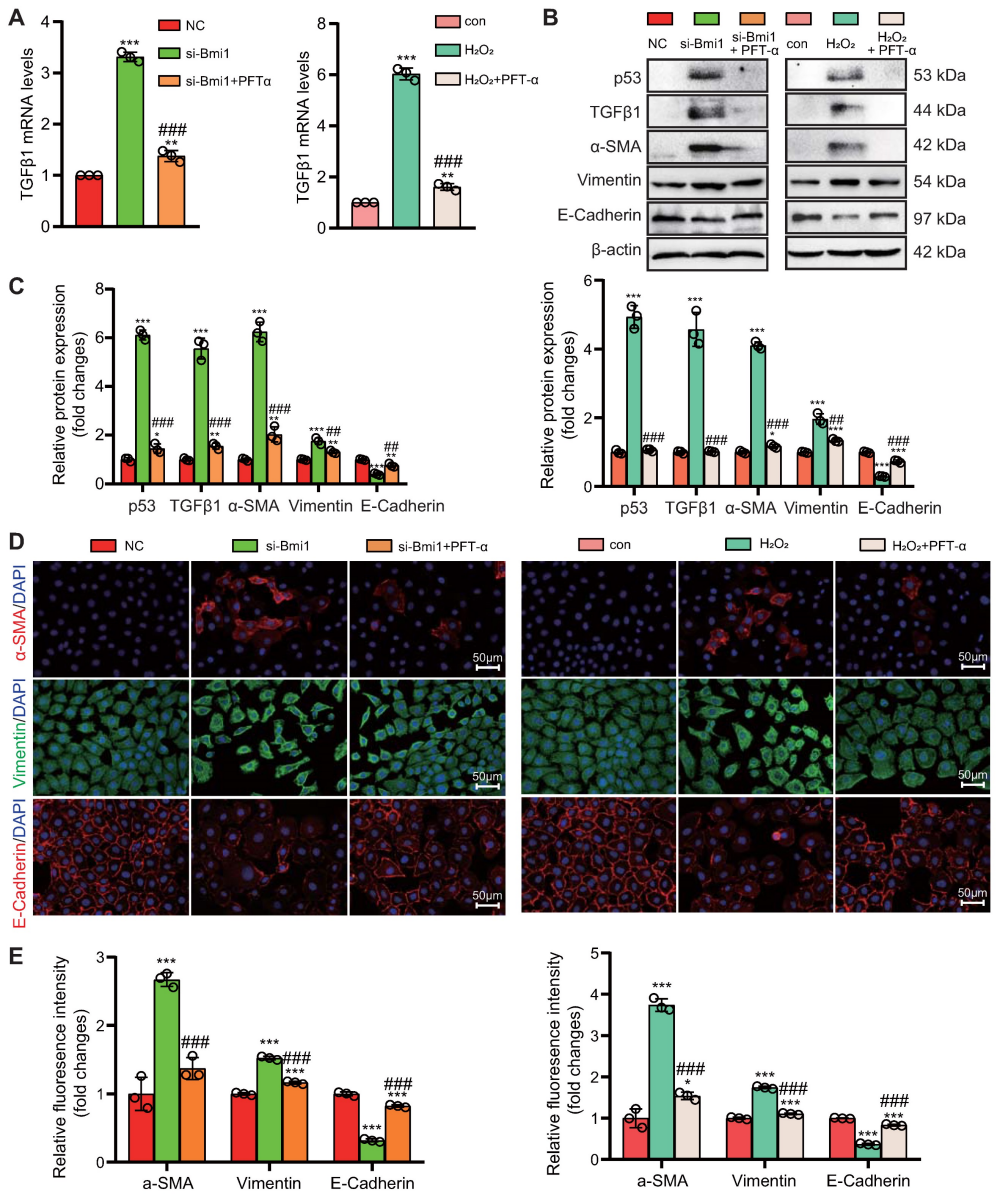
** Activation of the p53/TGFβ1 Signaling Pathway Promotes EMT.** si-Bmi1 transfection or H_2_O_2_ stimulation was performed in HK2 cells. **(A)** mRNA levels of TGFβ1 were detected.** (B & C).** Western blotting analysis of p53, TGFβ1, α-SMA, Vimentin, and E-Cadherin, with corresponding statistical analysis of relative protein expression levels.** (D)** IF staining for α-SMA, Vimentin, and E-Cadherin, with representative microscopic images, and** (E)** statistical analysis of relative fluorescence intensities. Three biological replicates were used per experiment. *: *P* < 0.05, **: *P* < 0.01, ***: *P* < 0.001, compared to the control group; ##: *P* < 0.01, ###: *P* < 0.001, compared to si-Bmi1-transfected or H_2_O_2_-treated cells.

To explore the role of the p53/TGFβ1 signaling pathway in promoting EMT, we employed two approaches: knocking down Bmi1 using siRNA and stimulating cells with H_2_O_2_ (80 μM, 48 hours) to elevate p53 protein levels in HK2 cells (Figs. 9C & D). Additionally, we selectively inhibited p53 protein levels and activity using the small molecule inhibitor PFT-α (Figs. 9C & D). We then assessed the impact of p53 on TGFβ1 mRNA and protein levels, as well as mesenchymal transition in HK2 cells, using qRT-PCR, IF, and Western blotting. The results demonstrated that, compared to the control group, HK2 cells with Bmi1 knockdown or those treated with H_2_O_2_ exhibited significant increases in TGFβ1 mRNA and protein levels, relative fluorescence intensities, and protein expression levels of mesenchymal markers, α-SMA and Vimentin (Figs. 9A-E). Conversely, the relative fluorescence intensity and protein expression levels of the epithelial marker, E-Cadherin, were significantly decreased (Figs. 9B-E). However, treatment with PFT-α (10 μM, 48 hours) partially reversed these changes in all the aforementioned parameters (Figs. 9A - E). These results conclusively indicate that the activation of the p53/TGFβ1 signaling pathway plays a pivotal role in driving EMT.

## Discussion

The present study provides novel insights into the link between Bmi1 deficiency and accelerated renal aging, highlighting the involvement of oxidative stress, DNA damage, and p53/TGFβ1-mediated EMT as key mechanisms. Our findings that Bmi1 expression declines with age in mouse kidneys and that Bmi1 knockout leads to oxidative stress, DDR, RTEC senescence, and fibrosis in kidneys are consistent with previous reports implicating Bmi1 as a vital protector against aging 32, 33. The amelioration of these aging-related changes by Chk2 knockout underscores the significance of DNA damage pathways in driving renal aging due to Bmi1 deficiency.

Notably, our results demonstrate for the first time that Bmi1 deficiency causes structural and functional impairment in mouse kidneys, evidenced by decreased glomerular number, cortex thickness, and kidney function, which could be partially restored by Chk2 knockout. These findings elucidate the importance of Bmi1 in maintaining renal structure and function with age. The increases in SCr, BUN, and UAL along with reduced SCrCl seen with Bmi1 knockout indicate development of kidney dysfunction, which correlates with human renal aging 40.

Additionally, our study provides novel evidence that Bmi1 deficiency leads to mitochondrial dysfunction and increased ROS production specifically in RTECs, inducing oxidative stress-mediated DNA damage. These results are consistent with previous research indicating that mitochondrial impairment and oxidative stress are central in initiating age-related kidney damage 41. Our findings that Chk2 knockout mitigates these effects emphasize the significance of DNA damage pathways in transducing oxidative stress into downstream aging sequelae. The p53 mainly maintains expression of genes that relate to anti-oxidant activity under physiological conditions 42. However, under severe and sustained stress, it promotes the expression of genes that relate to pro-oxidant activity 43. Our study found that Bmi1 deficiency caused a dramatic increase in p53 expression levels in the kidney tissues, but these levels reduced dramatically in Chk2 knockout mice, suggesting that Chk2 deletion may protect against oxidative stress that occurs in Bmi1 deficient kidney tissues by downregulating p53. However, the specific mechanism of p53's role in regulating oxidative stress in the kidney tissues remains to be investigated.

The promotion of RTEC senescence, SASP, and EMT by Bmi1 deficiency within kidneys and cultured RTECs is a key finding of our study. Cellular senescence has emerged as a driver of kidney aging 44, while SASP factors propagate senescence in a paracrine manner 45. Our results suggest Bmi1 is vital for suppressing senescence and SASP during kidney aging. Notably, the attenuation of EMT by Chk2 knockout implies that Bmi1 deficiency provokes EMT and renal fibrosis partly through DNA damage-induced cellular senescence.

A major novel finding of our study is the identification of TGFβ1 as a pivotal mediator of EMT upregulated by Bmi1 deficiency and inhibited by Chk2 knockout in RTECs. TGFβ1 is a major inducer of EMT and kidney fibrosis 46. Our results imply that Bmi1 suppresses TGFβ1 expression to attenuate EMT during kidney aging. Furthermore, we demonstrate for the first time that the upregulation of TGFβ1 by Bmi1 deficiency involves transcriptional activation by p53. As a central regulator of cell cycle arrest, senescence, and DDR, p53 is emerging as a key contributor to aging 47. Our findings elucidate a novel p53/TGFβ1 pathway underlying Bmi1 deficiency-induced EMT and kidney aging.

While this study enhances our understanding of mechanisms driving kidney aging, there are some limitations. Firstly, all experiments utilized mouse models, yet molecular drivers in mice may differ in humans. Validation in human kidney tissue samples is required before clinical translation. Additionally, further experiments should aim to elucidate the crosstalk between identified pathways and assess impacts on lifespan. Our focus was on early phenotypic changes, but examining later stage kidney disease development could reveal additional therapeutic windows. Moreover, we delineated an important functional role for p53; however, distinguishing transcriptional regulation of specific target genes from other p53 activities would further refine the mechanistic insights. Lastly, the efficacy and safety of proposed interventions like p53 or TGFβ1 inhibition require thorough preclinical testing prior to trials in humans. Despite these limitations, these findings open promising new avenues for detecting, preventing, predicting and slowing the progression of age-related kidney dysfunction. Clinical applications could include assessing p53 activation or TGFβ1 expression as early diagnostic biomarkers of renal aging. Our results also support exploring senolytic drugs to selectively eliminate senescent cells or TGFβ1/p53 signaling inhibitors as novel geroprotective agents that target root causes of kidney functional decline instead of just alleviating symptoms. This study elucidates crucial mechanisms underlying renal aging through an impressive array of techniques, identifies attractive targets for further drug development, and sets the foundation for future clinical translation efforts leveraging senescence and DNA damage pathways to predict, detect and treat age-related kidney disease.

In summary, our study provides several important advances. We demonstrate for the first time that Bmi1 deficiency drives mitochondrial and oxidative stress-induced DNA damage within kidneys, eliciting RTEC senescence, SASP, Chk2/p53/TGFβ1-mediated EMT, and kidney fibrosis. Our findings highlight Bmi1 as a crucial suppressor of renal aging that preserves mitochondrial and kidney function. We also elucidate DDR pathways, particularly p53, as key transducers of aging stimuli into downstream phenotypes. Finally, we identify TGFβ1 as a pivotal effector of EMT and kidney fibrosis induced by Bmi1 deficiency in a p53-dependent manner. Our study deepens understanding of mechanisms underlying renal aging and reveals novel targets to detect, prevent, and treat age-related kidney decline.

## Supplementary Material

Supplementary figures and table.

## Figures and Tables

**Table 1:** Primers used in this study for qRT-PCR are as follows.

Species	Name	Sequence	Tm (℃)	bp
Mouse	Chk2	S: 5'-TGACAGTGCTTCCTGTTCACA-3'	59.79	102
		AS: 5'-GAGCTGGACGAACCCTGATA-3'	58.89	
Mouse	TGFβ1	S: 5'-CTCCCGTGGCTTCTAGTGC-3'	60.15	133
		AS: 5'-GCCTTAGTTTGGACAGGATCTG-3'	58.73	
Mouse	gapdh	S: 5'-AGGTCGGTGTGAACGGATTTG-3'	60.88	123
		AS: 5'-TGTAGACCATGTAGTTGAGGTCA-3'	58.59	
Human	Bmi1	S: 5'-CATTGTCTTTTCCGCCCGCT-3'	61.59	143
		AS: 5'-AGTACCCTCCACAAAGCACAC-3'	60.20	
Human	Chk2	S: 5'-AGGGAAAGGAAAACGCCGTC-3'	60.89	179
		AS: 5'-TTACCTCTCCACAGGCACCA-3'	60.47	
Human	p53	S: 5'-TGGGACGGAACAGCTTTGAG-3'	60.25	89
		AS: 5'-CTCCCCTTTCTTGCGGAGAT-3'	59.46	
Human	TGFβ1	S: 5'-GCCCTGGACACCAACTATTGCT-3'	62.86	161
		AS: 5'-AGGCTCCAAATGTAGGGGCAGG-3'	64.36	
Human	gapdh	S: 5'-AAATCAAGTGGGGCGATGCT-3'	60.32	86
		AS: 5'-CAAATGAGCCCCAGCCTTCT-3'	60.32	
