# Supplementary material for: Chk2 Modulates Bmi1-Deficiency-Induced Renal Aging and Fibrosis via Oxidative Stress, DNA Damage, and p53/TGFβ1-Induced Epithelial-Mesenchymal Transition
Source: Int J Biol Sci. 2024 Mar 11;20(6):2008–26. doi: 10.7150/ijbs.93598 (PMC11008269; doi:10.7150/ijbs.93598)
Supplement: Supplementary file 1 — Supplementary figures and table. [file ijbsv20p2008s1.pdf]

Supplementary Figure 1

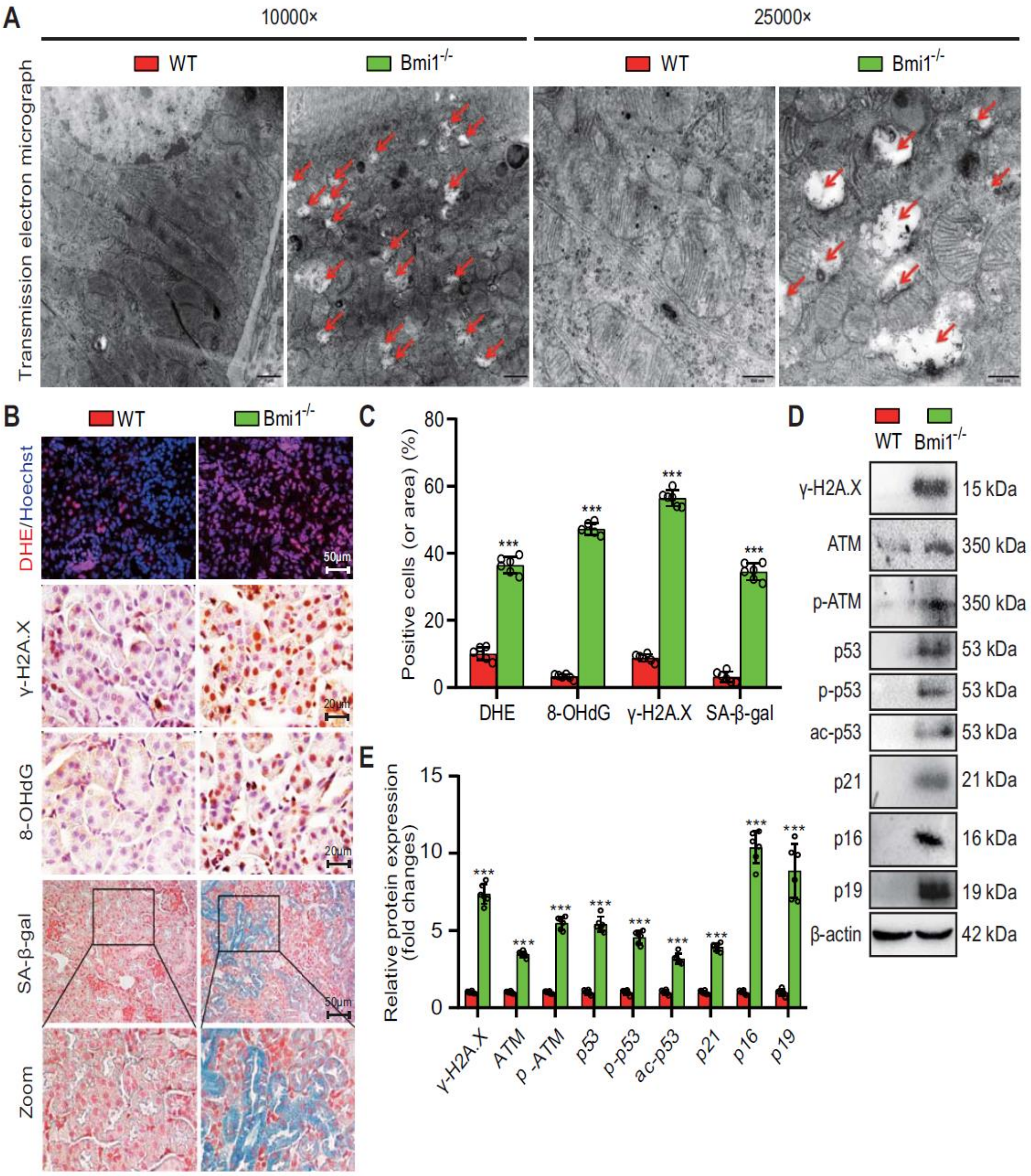

(A) Transmission electron micrograph of kidney tissues from 5-week-old littermate WT and *Bmi1*$^{-/-}$ mice, ↙: depicting mitochondrial vacuoles. **(B & C)** Micrographs depicting DHE fluorescence staining, γ-H2A.X and 8-OHdG immunohistochemical staining, and SA-β-gal histochemical staining, along with statistical graphs showing the percentage of positive cells (area) for each staining. **(D & E)** Western blotting images and statistical graphs illustrating the relative protein expression levels of γ-H2A.X, ATM, p-ATM, p53, p-p53, and ac-p53. Each experiment was conducted with 6 mice per group. Quantitative data are presented as mean ± standard deviation (Mean ± SEM). ***: $P < 0.001$, compared with 5-week-old WT mice.

Supplementary Figure 2

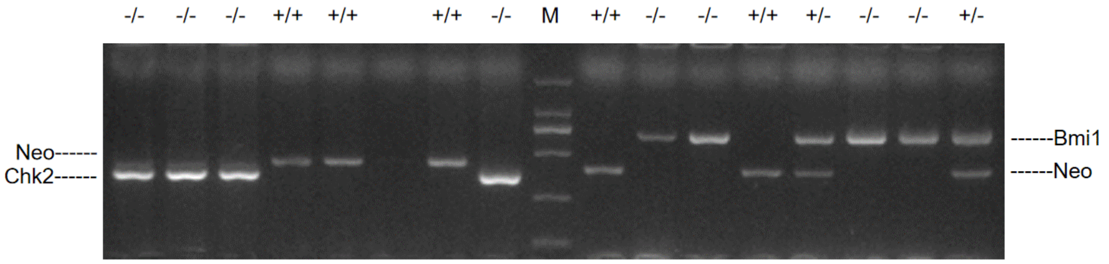

The gene band display of Bmi1 gene knockout mice shows that Bmi1$^{+/+}$ (wild-type) exhibits only one 687 bp band, Bmi1$^{-/-}$ (homozygous) exhibits only one 399 bp band, and Bmi1$^{+/-}$ (heterozygous) exhibits one 687 bp band and one 399 bp band.

The gene band display of Chk2 knockout mice shows that Chk2$^{+/+}$ (wild-type) exhibits only one 310 bp band, Chk2$^{-/-}$ (homozygous) exhibits only one 424 bp band, and Chk2$^{+/-}$ (heterozygous) exhibits one 310 bp band and one 424 bp band.

Supplementary Table 1

Name	WT_1	WT_2	WT_3	$Bmi1^{-/-}$_1	$Bmi1^{-/-}$_2	$Bmi1^{-/-}$_3	$Bmi1^{-/-}Chk2^{-/-}$_1	$Bmi1^{-/-}Chk2^{-/-}$_2	$Bmi1^{-/-}Chk2^{-/-}$_3
**Gm47283**	0.524302921	0.5459776	0.049121502	0.97209855	1.088367092	0.463273734	-0.577488062	-1.687509024	-1.378144313
**Grem2**	-1.210802927	-1.145109887	-1.311040095	0.243626811	0.307917012	0.160738006	1.416940987	1.027535873	0.51019422
**Trappc6a**	-1.743798489	-0.564267327	-0.433107587	1.242559248	1.579402412	-0.094296804	0.331389856	-0.509287754	0.191406444
**Col6a3**	-0.240012204	-1.50135635	-1.664659074	0.901837766	0.394119812	1.293945424	0.08536446	0.363867243	0.366892924
**Gm7897**	0.169873663	-1.740594292	-1.740594292	0.679973595	0.628663425	0.618161583	0.341001071	0.446402924	0.597112322
**Cd248**	0.194486584	-1.977796865	-1.274362571	0.843572172	0.661215169	0.935511194	-0.222413055	0.405665491	0.434121881
**Krt18**	-0.987032351	-1.299466724	-0.727217491	0.413849101	1.803194375	0.898553075	-0.603697301	0.041422752	0.460394564
**Ndn**	-1.01736766	-1.462218443	-0.629198048	1.078279489	1.451042997	0.734764222	0.17049041	-0.682878062	0.357085095
**Slfn2**	-1.057085818	-1.239617902	-1.218985525	1.047989011	1.242454424	0.606740599	-0.480334633	0.637275887	0.461563957
**Ncf2**	-0.195230624	-1.375762181	-1.448429057	1.51899082	1.049419224	0.639538621	-0.264171465	-0.163356206	0.239000869
**Clu**	-1.132925544	-1.311081469	-1.043952549	1.005429949	1.240931232	0.822213305	-0.304669196	-0.041657849	0.76571212
**P2rx2**	-1.761673116	-0.904271448	-0.712406135	1.407867508	0.752264745	0.987667067	-0.018008567	0.033220257	0.215339689
**Angpt4**	-1.968518199	-0.913147138	-0.577376759	1.182889146	0.735432818	0.753732253	0.216105819	-0.060881885	0.631763946
**Rec8**	-1.143194367	-1.459539248	-1.11872793	1.07030073	0.869730649	1.060472387	0.494904566	0.028639565	0.197413649
**Timp1**	-0.858491802	-1.627934382	-1.01932858	0.920830736	1.453709165	0.490465884	0.131807193	-0.081051346	0.589993134
**Adra2c**	-0.915361086	-1.413446559	-0.977985946	1.777454855	0.564145182	0.379051698	0.225037533	-0.304576997	0.665681321
**Pdgfa**	-0.552659249	-1.461800619	-1.001941106	1.794401146	0.807201533	0.539561389	-0.201539006	-0.37855281	0.455328723
**Lmcd1**	-0.314172698	-1.716429968	-1.06754741	1.553076947	0.875301978	0.623438539	0.189065566	-0.389719194	0.24698624
**Tgfb1**	-0.375315425	-1.809157735	-1.101633836	1.160202671	0.986268022	0.826292577	0.074955242	-0.291233838	0.529622321
**Ecm1**	-0.841535249	-1.595372108	-1.02429011	1.418107481	1.038914783	0.594936558	0.103205802	-0.1597409	0.465773741
**Twist1**	-0.855249774	-1.703497212	-0.876426596	1.413954293	0.897166102	0.878066087	0.12600612	0.048319489	0.07166149
**Hck**	-0.898368002	-1.862301058	-0.788568104	1.379410316	0.659104023	0.705786643	0.083903491	0.378129692	0.342902999
**Vegfc**	-1.740247208	-1.025021351	-0.980674056	1.048918832	0.645247286	1.037342076	0.39674284	0.213138956	0.404552625
**Nat8l**	-1.39948572	-0.745773228	-1.513725565	1.261923092	0.843924144	0.586429438	0.585757781	-0.064148161	0.445098219
**Pik3ip1**	-1.463441932	-1.086822785	-1.241520765	1.269902923	0.634558448	0.714903812	0.440257248	0.094496359	0.637666692
**Six2**	-1.243340419	-1.188433722	-1.218728601	1.29705496	1.037044172	0.708676688	0.236708162	-0.107353595	0.478372355
**Ifi202b**	-1.513663806	-0.585525461	-1.206510861	1.409719097	0.952348996	0.65157279	-0.346345087	-0.04669041	0.685094741
**Htra3**	-0.926021332	-0.738468364	-1.756434256	1.382254289	0.95796556	0.63631492	-0.190040971	0.173167022	0.461263131
